# Policy and practice suggestions to improve performance on the UNAIDS 90‐90‐90 targets: Results from a nominal group technique with HIV experts in Southwest Ethiopia

**DOI:** 10.1111/hex.13115

**Published:** 2020-08-05

**Authors:** Hailay Abrha Gesesew, Paul Ward, Kifle Woldemichael, Pamela Lyon, Lillian Mwanri

**Affiliations:** ^1^ Public Health Flinders University Adelaide SA Australia; ^2^ Epidemiology Mekelle University Mekelle Ethiopia; ^3^ Southgate Institute for Health, Society and Equity Flinders University Adelaide SA Australia

**Keywords:** ART in health post, ART in private clinic, HIV self‐testing, house to house HIV testing, nominal group technique, Policy

## Abstract

**Objective:**

This paper aims to evaluate the potential solutions to address negative outcomes of HIV care and treatment, that were proposed by HIV care providers, researchers and HIV programme managers in Southwest Ethiopia.

**Methods:**

A nominal group technique (NGT) was conducted with 25 experts in December 2017 in Jimma, Southwest Ethiopia. The NGT process included (a) an analysis of the previously qualitative study conducted with various Ethiopian HIV stakeholders who proposed possible solutions for HIV care and treatment; (b) recruitment of a panel of HIV experts in policy and practice to rate the proposed solutions in Ethiopia before a discussion (first round rating); (c) discussion with the panel of experts on the suggested solutions; and (d) conducting a second round of rating of proposed solutions. Content analysis and Wilcoxon signed rank test were applied to analyse the data.

**Results:**

Eighteen of the 25 invited panel of experts participated in the NGT. The following proposed solutions were rated and discussed as relevant, feasible and acceptable. *In order* of decreasing importance, the solutions were as follows: filling gaps in legislation, HIV self‐testing, the *teach‐test‐link‐trace* strategy, house‐to‐house HIV testing, community antiretroviral therapy (ART) groups, providing ART in private clinics and providing ART at health posts.

**Conclusions:**

The current study findings suggested that, to address HIV negative outcomes, priority solutions could include mandatory notification of partner's HIV status, HIV self‐testing and the involvement of peer educators on the entire HIV care programme.

## 1 INTRODUCTION

The human immunodeficiency syndrome (HIV) care cascade comprises HIV testing and diagnosis, assessment for antiretroviral therapy (ART) eligibility and treatment, patient retention and virological suppression.^1^, ^2^ The discovery of ART in 1996 has transformed the lives of tens of millions of individuals with HIV.^3^ Nevertheless, research has identified a growing number of challenges that mask the benefits of ART, such as late HIV diagnosis and poor linkage to ART care,^4^, ^5^ lost to follow up,^6^, ^7^ poor ART compliance ^8^, ^9^, ^10^ and virological failure.^11^, ^12^, ^13^ These challenges make it difficult to attain the Joint United Nations Program on HIV and AIDS (UNAIDS) 90‐90‐90 targets which relate, respectively, to the proportion of HIV patients: (a) who know their HIV status, (b) who are on ART and (c) who are on ART and have viral load suppressed.^14^, ^15^, ^16^

Across the globe, countries have designed different approaches to improve performance against the 90‐90‐90 targets. Although Ethiopia has endorsed the UNAIDS targets,^17^ progress towards these are yet to be formally evaluated, and performance improvement strategies remain unexplored. Our evaluation of the performance of HIV care in Southwest Ethiopia against the UNAIDS set targets, using surrogate measures from 12 years of retrospective data (2003‐2015) provided a score of 35‐66‐65,^18^ indicative of a lower than the UNAIDS expected performance. Evidence revealed that barriers to early HIV care presentation include low education, unemployment, rural residence, low income, comorbidities, injectable drug use (IDU) and excessive alcohol consumption.^19^, ^20^ Furthermore, barriers to retention in care include rural residence, low education, poor baseline functional status, advanced baseline WHO clinical stage and poor CD4 counts.^21^, ^22^, ^23^, ^24^

To explore contextual facilitators, barriers to and solutions for HIV care and treatment in Ethiopia, we employed a qualitative interview with HIV patients, HIV health‐care providers, community advocates participating in HIV care, and HIV care system administrators.^25^ The facilitators identified in the qualitative paper^25^ included improved knowledge and trust in ART care, supportive environment from family, provider‐initiated HIV counselling and testing services (PICT), and voluntarily HIV testing and counselling services (VCT). The qualitative paper^25^ also identified the following barriers that led to poor HIV care and treatment outcomes: HIV stigma, poor knowledge and trust in ART care, poor access and availability to ART care services, direct and associated cost of ART, structural factors resulting from patriarchal society, alternative perspectives of traditional healers and fragmented health‐care system. As described above, to address these barriers, the qualitative paper reported a set of proposed solutions to improve HIV policy and practice in Ethiopia.^25^

This paper is an extension of our previous qualitative paper.^25^ Following the qualitative study,^25^ we undertook a nominal group technique (NGT) to rank the relevance, feasibility and acceptability of the proposed solutions receiving expert advice from key stakeholders. These experts comprised: academics, service providers and HIV programme managers. The aim of the current paper is to report on a consensus development study, using the NGT, which attempted to rank these solutions. The NGT outcomes are expected to inform measures to enhance and strengthen HIV care practices, guide future HIV policy development and generate new research ideas for further studies to improve Ethiopia's performance to the UNAIDS targets.

## 2 METHODS

### 2.1 Nominal Group Technique (NGT) study design

The present study used the NGT, a design for consensus development,^26^, ^27^, ^28^ to rank the proposed solutions provided by our qualitative paper^25^ and to improve HIV care and treatment in Ethiopia. As a planning technique, NGT involves four main steps: (a) silent generation of idea(s), (b) a round‐robin, (c) clarification and voting and (d) ranking or rating.^29^ The NGT can be modified, for example, by generating ideas initially from a literature review or an exploratory study instead of ‘*silent generation*’.^30^, ^31^ Box 1 presents the details of the modified NGT process employed for the current study.

Box 1 **The Nominal Group Technique process**

**Definition of Problem** →→ which of the suggested solutions are relevant, feasible and acceptable?

**Selection of experts** →→ e‐mail sent to potential experts that involve HIV workers from hospital and health centre, HIV programme coordinators from local and global non‐governmental organizations, town health office and zonal health departments, and researchers from university.

**First round of nominal group** →→ participants rated each suggested solution using three criteria of importance—relevance, feasibility and acceptability—on scale from 1 ‘agree’ to 3 ‘disagree’. Participants also asked to suggest additional solutions for HIV diagnosis, ART linkage and retention. The facilitators summarized the results and calculate a score.

**Second round of nominal group** →→ the facilitators present solutions for HIV care and treatment suggested from a qualitative inquiry. Then after, the participants discussed and re‐rated the solutions.

**Results analysed for agreement using predefined rules** →→ the facilitators summarized the final statement of consensus including the minority suggestions.

(Adapted from Jones & Hunter, 1995)

HIV: human immunodeficiency virus.

### 2.2 Size and selection of NGT expert participants

Guided by Chitu and colleagues,^32^ appropriate experts were identified and invited via e‐mail to attend the NGT. We utilized consensus development techniques provided elsewhere,^28^, ^33^ for the definition of an ‘expert’. For experts to participate, we: (a) prepared a list of relevant disciplines and organizations before nominating names of the experts; (b) developed a list of potential experts from the identified disciplines and organizations described in step one; (c) contacted experts listed in step 2 and requested them to nominate other experts; (d) ranked experts listed in step 2 and 3 according to their expertise, disciplines and organization; and (e) invited experts in order of their ranking until the targeted sample size was achieved. Twenty‐five (25) participants—including HIV programme managers, researchers and service providers, were invited to participate in the NGT, and only 18 accepted the invitation to participate.

#### 2.2.1 NGT idea generation process

We modified the ‘*silent generation*’ step by obtaining the ideas from our previous qualitative paper^25^ and from further suggestions made by the participants of the NGT process. From the findings of our qualitative paper, the following seven potential solutions related to HIV testing and treatment were proposed. These included (a) HIV self‐testing, (b) house‐to‐house HIV testing, (c) the peer educators‐led *teach‐test‐link‐trace* strategy, (d) ART provision in private clinics, (e) community ART groups, (f) ART provision in health posts and (g) filling the gaps in the legislation. Box 2 describes the definitions of these interventions.

Box 2 **Operational definitions of seven interventions for HIV care and treatment and three criteria of prioritization**

The operational terms used for the seven suggestions to improve HIV care and treatment are defined as follows.

- *HIV self‐testing*: refers to a screening process whereby a person who wants to know one's HIV status collects a specimen, perform a test and interpret the result in private.
- *House‐to‐house HIV testing*: refers to conducting HIV testing in every house by HEWs or trained lay counselors or peer educators—this process includes collecting a specimen, performing a test, interpreting the result and referral for further follow‐up test or linkage (if the result is positive).
- *Teach‐test‐link‐trace strategy*: involves formally employing and assigning of peer educators (*HIV + persons disclosed themselves publicly*) with HEWs (health extension workers) to teach the community about HIV, conduct HIV testing, linking into ART care and trace lost patients coined as *Teach‐test‐link‐trace strategy* (TTLT).
- *ART in private clinics*: comprises the provision of ART care in private health clinics by the health workers employed in the clinic. ART will be provided for free by the government.
- *Community ART groups*: refers to a process whereby stable HIV + persons (who disclose publicly) living in near places establish a group and take their medications turn by turn or in rotation. They choose a leader who arranges monthly meeting to count pills and check the overall ART adherence. The people on ART will be told to come to the clinic whenever they feel ill.
- *ART in health posts*: involves the provision of ART in health post by HEWs. Health post is a grassroot level of primary health‐care structure in Ethiopia with two community health workers (named health extension workers) serving on average 5000 people.
- *Filling gaps in legislation*: refers the need of legislation to suing an HIV + man who does not disclose his status to his wife after repeated counselling (as this prevents the woman from timely engagement to HIV care and prevent HIV transmission to child (if pregnant)) or vice versa. Another scenario is the need of legislation to suing religious leaders or witch doctors who declare HIV cure while not—as this is a false witness and against legislation of the nation. In addition, if the religious leaders or witch doctors tell patients to throw the pills and if patients die or sick seriously as a result of this, he/she is responsible to the death or attempt, and this is against legislation of the nation.

The operational criteria used during NGT to rate or evaluate proposed suggestion in a self‐ administered questionnaire included:

- Relevance: refers to a scientific or societal benefit of a specified intervention to the context of HIV diagnosis, treatment linkage and retention in care.
- Feasibility: refers to technical, logistical, cultural (including religious) and legal considerations to implement a specified intervention.
- Acceptability: refers how well is received by the target population considering attitude, burden, effectiveness, ethics and opportunity cost.

### 2.3 NGT discussion and ranking of solutions process

The process of the current NGT study was facilitated by the three study authors (HAG, KW and LM). Participants were asked to rate or evaluate each suggestion based on criteria of relevance, feasibility and acceptability (Box 2) using a self‐administered questionnaire. The questionnaire had a three‐point Likert scale as follows: agree (1), neural (2) and disagree (3), and had a free space for explanatory remarks. Two rounds of rating were conducted by the experts. For the initial rating round (R‐1), experts were asked to rate the seven suggested solutions obtained from the qualitative study.^25^ Guided by the following question, ‘*What new programs do you suggest to improve HIV diagnosis, ART linkage or ART retention apart from these suggested solutions?’* the NGT participants provided additional suggestions based on their expertise. The old and new suggestions were merged, but the new not changed the number of potential solutions to be ranked. After evaluation of the R‐1 rating, a rigorous discussion was held. Participants were asked to re‐rate (R‐2) the solutions and provide justification if they did differ significantly with group opinion. After completion of the rating, facilitators collected the rating as part of data. The entire NGT study data comprised the quantitative component derived from NGT rating and the qualitative component comprising the audio‐recording of the NGT discussions, the notes made from the justifications remarks and researchers’ notes made during the NGT processes. Data were analysed as described below.

### 2.4 NGT data management and analyses

We applied descriptive and inferential statistics to analyse the quantitative component of the NGT. To examine the statistical difference between the ratings in R‐1 and R‐2, we used a Wilcoxon signed rank test.^34^ Thus, 21 hypotheses (7*3, ie seven suggested solutions and three criteria for each) were tested. The hypotheses were as follows:

H_A_ (alternative hypothesis): There was a difference in rating of the suggested solutions between rounds 1 and 2.

The qualitative data were analysed using content analysis^35^ in six steps as follows. We: (a) transcribed audio‐recording, notes from justification of made remarks and field notes, (b) familiarized with data through repetitive reading of the transcripts, (c) identified, listed notes (codes) and tallied the codes, (d) indexed the nodes and classified them, (e) read through the classification to name each theme and (f) reviewed the steps from a‐e to map, interpret and confirm that the categories were exhaustive, mutually exclusive and independent.^35^

## 3 RESULTS

Eighteen (18) members of the 25 identified and invited panellists participated in the discussion making a 72% acceptance rate. Of all the experts, three were full professors (Professors of Epidemiology, Public Health and Reproductive Health), four were assistant professors (Epidemiology, Health Service Management and Mental Health), and five were lecturers.^36^ All panel members took part in Round‐1 (R‐1) rating and the discussion; 16 members participated in Round‐2 (R‐2) rating. Two experts, a clinician and a programme coordinator, dropped out from the discussion due to unforeseen emergency commitments. Tables 1 and 2 describe individual rating of proposed measures for improving HIV care and treatment in R‐1 and R‐2, respectively.

**Table 1:** Relevance, feasibility and acceptability of suggested solutions for improving HIV care and treatment (Round 1)

P	Sex	Age (y)	Round 1 (before discussion; 9:30 AM) on 21 December 2017; Jimma Central Hotel, Jimma, Southwest Ethiopia																				
			P = Participants R = relevant; F = feasible; A = acceptable; 1 = Agree; 2 = Neutral; 3 = Disagree																				
			HIVST			H2H			TTLT			ART_HP_			ART_PC_			CAG			**Legislation**		
			R	F	A	R	F	A	R	F	A	R	F	A	R	F	A	R	F	A	**R**	**F**	**A**
P1	‐	‐	1	1	1	1	2	1	2	2	2	1	1	1	3	3	1	1	1	1	2	2	2
P2	M	39	1	2	2	1	2	2	1	1	1	3	3	3	3	3	3	1	2	2	1	1	1
P3	M	30	1	3	1	2	3	2	1	3	3	1	3	1	1	1	1	1	1	1	1	1	1
P4	F	53	1	3	2	1	3	2	1	3	2	1	1	3	1	1	1	1	1	2	1	2	2
P5	M	60	1	1	2	1	1	2	2	2	2	1	1	1	1	1	1	1	1	2	1	1	1
P6	M	48	1	1	1	1	1	2	1	1	1	3	3	3	1	1	1	2	2	2	1	1	1
P7	M	58	3	3	3	1	1	1	2	2	2	3	3	1	1	1	1	2	2	2	2	1	2
P8	M	30	1	1	1	1	1	2	1	1	1	1	1	3	1	1	1	1	2	2	1	1	1
P9	F	48	1	1	1	2	2	2	1	1	1	2	2	2	1	1	1	3	3	3	1	1	1
P10	M	32	1	3	2	1	3	3	1	1	1	1	2	1	1	1	1	1	1	1	1	1	1
P11	M	63	1	1	2	1	2	1	1	1	3	1	1	1	1	2	1	1	1	1	1	1	1
P12	M	38	1	1	2	1	2	1	1	1	1	1	1	1	1	1	1	1	2	1	1	1	1
P13	M	31	3	3	3	2	2	2	1	1	1	3	3	3	2	2	2	1	1	1	1	1	1
P14	M	30	1	2	2	1	2	2	1	2	2	2	1	2	2	1	2	2	1	2	1	2	2
P15	F	45	1	2	1	1	2	3	1	3	3	1	2	1	1	1	1	1	3	3	1	1	1
P16	M	45	1	1	1	1	1	2	2	2	3	1	2	3	1	1	1	3	3	3	1	1	1
P17	M	33	1	1	1	1	1	1	1	1	1	3	3	3	3	3	3	1	1	1	1	1	1
P18	M	29	1	3	2	1	3	1	1	1	1	1	1	2	1	3	2	1	3	1	1	3	3
**Sum**		**1**	**16**	**9**	**8**	**15**	6	6	**14**	**10**	**9**	**11**	**8**	**8**	**13**	**12**	**13**	**13**	**9**	**8**	**16**	**15**	**13**
		**2**	0	3	**8**	3	**8**	**10**	4	5	5	2	4	3	2	2	3	3	5	7	2	2	4
		**3**	1	6	2	0	4	2	0	3	4	5	6	7	3	4	2	2	4	3	0	1	1

Note

HIVST is a process whereby a person who wants to know one's HIV status collects a specimen, perform a test and interpret the result in private—this is a screening test. H2H refers to conducting HIV testing in every house by HEWs or trained lay counsellors or peer educators—this process includes collecting a specimen, performing a test, interpreting the result and referral for further follow up test or linkage (if the result is positive). TTLT involves formally employing and assigning of peer educators (*HIV + persons disclosed themselves publicly*) with HEWs (health extension workers) to teach the community about HIV, conduct HIV testing, linking into ART care and trace lost patients coined as *Teach‐test‐link‐trace strategy* (TTLT). ARTHP is the provision of ART in health post by HEWs. ARTPC is the provision of ART care in private health clinics by the health workers employed in the clinic. ART will be provided for free by the government. CAGs is a process whereby stable HIV + persons (who disclose publicly) living in near places establish a group and take their medications turn by turn or in rotation. They choose a leader who arranges monthly meeting to count pills and check the overall ART adherence. The people on ART will be told to come to the clinic whenever they feel ill. Legislation refers the need of legislation to suing an HIV + man who does not disclose his status to his wife after repeated counselling (as this prevents the woman from timely engagement to HIV care, and prevent HIV transmission to child (if pregnant)) or vice versa. Another scenario is the need of legislation to suing religious leaders or witch doctors who declare HIV cure while not—as this is a false witness and against legislation of the nation. In addition, if the religious leaders or witch doctors tell patients to throw the pills and if patients die or sick seriously as a result of this, he/she is responsible to the death or attempt, and this is against legislation of the nation.

**Table 2:** Relevance, feasibility and acceptability of suggested solutions for improving HIV care and treatment (Round 2)

P	Round 2 (after discussion; 2:30 PM) on 21 December 2017; Jimma Central Hotel, Jimma, Southwest Ethiopia																				
	P = participants; R = relevant; F = feasible; A = acceptable; 1 = Agree; 2 = Neutral; 3 = Disagree																				
	HIVST			H2H			TTLT			ART_HP_			ART_PC_			CAG			Legislation		
	R	F	A	R	F	A	R	F	A	R	F	A	R	F	A	R	F	A	R	F	A
P1	1	2	3	3	2	3	1	1	1	3	3	3	3	2	3	1	1	1	1	1	1
P2	1	2	2	1	2	2	1	1	2	2	2	2	3	3	3	2	2	2	1	1	1
P3	1	1	1	1	1	1	2	2	2	2	2	2	3	3	3	2	2	2	1	1	1
P4	1	3	1	2	3	3	1	2	3	1	3	2	1	1	1	1	1	1	1	1	1
P5	1	1	2	1	1	2	1	2	3	1	1	1	2	2	2	1	2	2	2	1	2
P6	1	1	1	1	1	1	1	1	1	3	3	3	3	3	3	1	1	1	1	1	1
P7	1	2	1	1	1	1	2	2	1	1	2	1	1	1	2	2	1	2	1	1	1
P8	1	1	1	1	1	2	1	1	1	1	1	3	1	1	1	1	1	1	1	1	1
P9	2	2	2	2	2	2	1	1	1	1	2	2	1	1	2	1	2	2	1	1	1
P10	1	3	1	1	1	3	1	1	2	1	2	1	1	1	1	1	3	2	1	1	2
P11	1	1	1	1	1	1	1	2	2	1	2	2	1	2	2	1	2	2	1	2	2
P12	1	3	2	2	2	2	1	3	2	1	2	2	1	1	1	1	1	1	1	1	1
P13	1	3	2	1	1	2	1	1	3	1	1	1	2	1	1	1	1	1	1	2	1
P14	2	3	1	2	1	2	2	2	1	2	2	1	2	1	2	1	2	2	2	2	1
P15	1	1	1	2	2	2	1	1	1	2	2	2	3	3	3	2	2	2	1	1	1
P16	1	3	3	1	3	1	1	3	1	2	3	1	1	2	3	2	3	1	1	1	1
1	**14**	**6**	**9**	**10**	**9**	5	**13**	**8**	**7**	**9**	3	6	**8**	**8**	5	**11**	**7**	7	**14**	**13**	**13**
2	2	4	5	5	5	**8**	3	6	6	5	**9**	**7**	3	3	**7**	5	**7**	**9**	2	3	3
3	0	6	2	1	2	3	0	2	3	2	4	3	5	5	4	0	2	0	0	0	0

Note

HIVST is a process whereby a person who wants to know one's HIV status collects a specimen, perform a test and interpret the result in private—this is a screening test. **H2H** refers to conducting HIV testing in every house by HEWs or trained lay counsellors or peer educators—this process includes collecting a specimen, performing a test, interpreting the result and referral for further follow‐up test or linkage (if the result is positive). TTLT involves formally employing and assigning of peer educators (*HIV + persons disclosed themselves publicly*) with HEWs (health extension workers) to teach the community about HIV, conduct HIV testing, linking into ART care and trace lost patients coined as *Teach‐test‐link‐trace strategy* (TTLT). ARTHP is the provision of ART in health post by HEWs. ARTPC is the provision of ART care in private health clinics by the health workers employed in the clinic. ART will be provided for free by the government. CAGs is a process whereby stable HIV + persons (who disclose publicly) living in near places establish a group and take their medications turn by turn or in rotation. They choose a leader who arranges monthly meeting to count pills and check the overall ART adherence. The people on ART will be told to come to the clinic whenever they feel ill. Legislation refers the need of legislation to suing an HIV + man who does not disclose his status to his wife after repeated counselling (as this prevents the woman from timely engagement to HIV care, and prevent HIV transmission to child (if pregnant)) or vice versa. Another scenario is the need of legislation to suing religious leaders or witch doctors who declare HIV cure while not—as this is a false witness and against legislation of the nation. In addition, if the religious leaders or witch doctors tell patients to throw the pills and if patients die or sick seriously as a result of this, he/she is responsible to the death or attempt, and this is against legislation of the nation.

After calculating the average score of relevance, feasibility and acceptability, in R‐1, the seven measures were recommended *in the following order*: (a) legislation, (b) ART in private clinics, (c) HIV self‐testing, (d) *teach‐test‐link‐trace* strategy, (e) community ART groups, (f) ART in health post and (g) house‐to‐house HIV testing. In R‐2, following discussion and recalculation, the proposals, in their average score, were re‐ranked as follows: (a) legislation, (b) HIV self‐testing, (c) *teach‐test‐link‐trace* strategy, (d) house‐to‐house testing, (e) community ART groups, (f) ART in private clinics and (g) ART in health posts. In both rounds, legislation and self‐testing were among the top three recommended solutions, while ART in health post was among the least popular suggestions. Following the discussion, the *teach‐test‐link‐trace* strategy moved up from fourth to third place and house‐to‐house HIV testing moved up from seventh to fourth place. Meanwhile, ART in private clinics dropped from second to sixth place. The Wilcoxon signed rank test showed no statistical difference between R‐1 and R‐2 for all suggestions (Table 3). In the open discussion, all of the suggested measures were discussed, and expert panels have forwarded directions on how these programmes can be implemented. Below, we have summarized the findings with the suggested solutions.

**Table 3:** Wilcoxon Signed Rank Test output for HIV care and treatment solutions, n = 16

Solution	Criteria	Round				Percentiles			Test^a^ (*P*‐value)
			N	Mean	SD	25th	50th	75th	
HIVST	Relevance	Round 1	16	1.00	0.000	1	1	1	.317
		Round 2	16	1.06	0.250	1	1	1	
	Feasibility	Round 1	16	1.63	0.719	1	1.5	2	.194
		Round 2	16	1.94	0.929	1	2	3	
	Acceptability	Round 1	16	1.56	0.629	1	1.5	2	.763
		Round 2	16	1.63	0.619	1	2	2	
H2H	Relevance	Round 1	16	1.27	0.594	1	1	1	1.000
		Round 2	16	1.27	0.458	1	1	1	
	Feasibility	Round 1	16	1.67	0.724	1	2	2	.234
		Round 2	16	1.93	0.799	1	2	3	
	Acceptability	Round 1	16	1.67	0.816	1	1	2	.305
		Round 2	16	1.93	0.704	1	2	2	
TTLT	Relevance	Round 1	16	1.13	0.342	1	1	1	1.000
		Round 2	16	1.13	0.342	1	1	1	
	Feasibility	Round 1	16	1.44	0.629	1	1	2	.417
		Round 2	16	1.63	0.806	1	1	2	
	Acceptability	Round 1	16	1.69	0.793	1	1.5	2	1.000
		Round 2	16	1.69	0.793	1	1.5	2	
ART_HP_	Relevance	Round 1	16	1.63	0.806	1	1	2	.527
		Round 2	16	1.50	0.632	1	1	2	
	Feasibility	Round 1	16	1.81	0.834	1	2	2.75	.608
		Round 2	16	1.94	0.680	1.25	2	2	
	Acceptability	Round 1	16	2.06	0.854	1	2	3	.564
		Round 2	16	1.94	0.772	1	2	2.75	
ART_PC_	Relevance	Round 1	16	1.56	0.892	1	1	2.75	.180
		Round 2	16	1.75	0.931	1	1	3	
	Feasibility	Round 1	16	1.69	0.873	1	1	2.75	.317
		Round 2	16	1.56	0.892	1	1	2.75	
	Acceptability	Round 1	16	1.75	0.856	1	1.5	2.75	.480
		Round 2	16	1.63	0.885	1	1	2.75	
CAGs	Relevance	Round 1	16	1.31	0.602	1	1	1.75	.480
		Round 2	16	1.19	0.403	1	1	1	
	Feasibility	Round 1	16	1.75	0.775	1	2	2	.414
		Round 2	16	1.63	0.719	1	1.5	2	
	Acceptability	Round 1	16	1.56	0.629	1	1.5	2	.564
		Round 2	16	1.50	0.632	1	1	2	
Legislation	Relevance	Round 1	16	1.00	0.000	1	1	1	.083
		Round 2	16	1.19	0.403	1	1	1	
	Feasibility	Round 1	16	1.25	0.577	1	1	1	.577
		Round 2	16	1.38	0.619	1	1	2	
	Acceptability	Round 1	16	1.31	0.602	1	1	1.75	.739
		Round 2	16	1.38	0.500	1	1	2	

^a^ Test refers to Wilcoxon signed rank test; ARTHP, antiretroviral therapy (ART) in health post; ARTPC, ART in private clinic; CAGs, community ART groups; H2H, house‐to‐house HIV testing; HIVST, HIV self‐testing; legislation, filling gaps in legislation; N, number of participants; SD, standard deviation; TTLT, teach, test, link and trace strategy.

### 3.1 Filling the gaps in the legislation (Legislation)

The participants strongly agreed that new legislation is needed to improve HIV care and treatment performance. A few gaps in the law were identified. For example, currently, husbands could choose not to disclose their HIV status to their wives, which could affect women and unborn children. Likewise, wives are under no legal obligation to disclose their HIV status to their husbands. Therefore, the expert panel suggested a legal requirement for mandatory disclosure of one's HIV status to a partner, or a combination of a new legal framework and public health promotion of voluntary notification. An Assistant Professor of Psychiatry supported this.

……. The same is true with HIV disclosure. There must be a legal framework that obligate partner to disclose their (HIV) status. Otherwise, the harm will be beyond the individual partners. For example, it is a risk to the coming baby. There should be a legislation…… (Participant 8)

Another gap that expert mentioned was an absence of laws that addresses traditional healers or religious curers who declare they have cure to HIV and/or direct HIV patients to discard ART medications. It was noted that if patients discarded ART pills on traditional or religious grounds, they would jeopardize not only their own survival but also the health and survival of others. It is a common knowledge that non‐compliance to the ART regime may lead to drug resistance. To address the above‐mentioned problems, the expert panel recommended a legislative framework that would make traditional healers and/or religious curers responsible for their actions. For example, the Assistant Professor of psychiatry said,

Collaboration with religious leaders, it is good and bad. It is good because patients can get psycho‐social and spiritual supports. But if we can come to treatment and cure, the religious fathers are not good. They should not declare that somebody is cure from HIV, and the government should develop a rule to sue them. (Participant 8)

Conversely, other experts were reluctant to impose a legislation on religious or traditional healers. They stated that it may result in a reverse effect on the HIV care continuum, since the religious leaders may pass their blessing to the mass but it is up to the interest of the individual patient to accept the blessing and decide. Instead, the participants advised raising awareness of HIV patients, religious leaders or traditional healers and the community at large about the above issues. Alternatively, it was suggested that there was a need to design a positive reinforcement strategy. For example, rather than prosecuting, or enforcing rules and regulations, a certificate could be provided as an incentive to partners who notify their HIV status voluntarily.

### 3.2 HIV self‐testing (HIVST)

The experts acknowledged that HIV self‐testing (HIVST) reduces HIV‐related stigma. For these measures to be effective and complementary, experts also suggested a combination of HIV self‐testing and facility‐based HIV counselling. It was recognized that patients could receive counselling in the health facility but do the self‐testing wherever they wanted. According to the experts, the HIVST programme would limit HIV transmission and promote early presentation to HIV care. An Assistant Professor of Epidemiology said,

With the issue of HIV self‐testing, why don’t you do the combination? For example, a patient can get counseling service in a clinic but can employ the testing (HIV) by him/her self where ever he wants…. it avoids the minuses of HIV self‐testing you have mentioned… (Participant 3)

Furthermore, the expert panel suggested that an appropriate testing system based on special population, age and level of education could be developed to meet needs of at risk populations such as commercial sex workers, long‐distance truck drivers, factory workers and mobile merchants. However, implementing this programme would have some limitations such as fear of not coping with positive result, attempting suicide, infecting others deliberately and not linking to care after being positive.

### 3.3 Teach‐Test‐Link‐Trace strategy (TTLT)

In the past, HIV was thought to be a death sentence. However, because of the involvement of HIV‐positive patients themselves as peer educators, the HIV fear factor is reducing. In hospitals and health centres, as an expert noted, it is becoming customary to send a new HIV patient to peer educators for counselling before putting them on a treatment plan. As a result, the impact of peer educators has been positive in reducing HIV‐related stigma and loss to follow up. In addition, peer educators have significantly improved ART adherence and the retention in care. A Reproductive Health Professor said,

I like HIV patients to be involved into the care. There are a lot of volunteers … However, due to resources issue, they may stop. We see this in X health center. So, get ready for the resources and involve patients. We are seeing the benefit live. (Participant 4)

Peer educators also conduct home‐based HIV testing for families or persons who do have contact with known HIV patient(s), a process known as index family testing. Thus, it was suggested that we could upgrade them to conduct a routine home‐based HIV testing whether families do or do not have contact with HIV‐positive person. Participants suggested that the use of peer educators in HIV care is important for the following reasons: (a) they themselves are HIV positive and can easily convince people to test for HIV via sharing of their experiences; (b) they are known to have high capability of maintaining confidentiality and people will trust them better compared to health extension workers (HEWs); and (c) they can also secure a job from the program. Nevertheless, the experts described that the issue of logistics may limit the implementation of such a programme.

### 3.4 House‐to‐House HIV testing

The majority of panel members agreed that house‐to‐house HIV testing is not easily feasible or acceptable. For example, the experts noted, if the house‐to‐house HIV testing is carried out using HEWs, it will be less acceptable for the following reasons; (a) HEWs might break confidentiality since most of them, particularly rural ones, are recruited from the community members they are serving; (b) HEWs are overburdened and are known to regularly complain of job dissatisfaction; and (c) they have low capacity to monitor the clinical characteristics of HIV patients. It was also noted that significant resources would be required should such a plan scale up nationally, questioning its feasibility. The fear of stigma that would emanate from the family could also discourage HIV testing in homes. This was noted as a significant hurdle by Associate Professor of Reproductive Health,

I don't think HEWs are capable at all. It will be a novel complication to the HIV care if we involve them in the HIV testing program—the confidentiality, our society’s reservation, etc By the way the house‐based testing is logistically impossible. (Participant 5)

Although less feasible and acceptable, the programme was rated relevant. In this regard, the experts acknowledged some benefits would include increasing access to community, that is, services to be at home, and in some ways, it might reduce HIV‐related stigma. It was also suggested that this programme can also be integrated with HIV self‐testing.

### 3.5 Community ART groups

The experts rated the community ART groups programme relevant but less acceptable and feasible for the following reasons: (a) it was feared that people may not disclose their status; and (b) it was noted that ART could be abused, that is sold or exchanged by the group of leaders, leading to non‐adherence and drug resistance.

However, some experts noted that the programme is novel and suggested that it may be implemented in rural areas, since many people in rural areas travel long distances (hundreds of kilometres) to get in to ART services. Furthermore, the programme may help HIV group members to establish their own social networks. For example, an Associate Professor of Reproductive Health said,

… community ART group is so novel, especially to our society—our people travel hundreds of kilometers to get ART services. If we have people who disclose their HIV status, they can take the pills turn by turn. This is fantastic. (Participant 5)

### 3.6 ART in Private Clinics

The panel members had major reservations to providing ART in private clinics for the following reasons including:(a) a potential to misuse ART drugs, (b) treatment could be expensive due to high costs associated with HIV care and (c) the likelihood of failure of private clinics allocating enough time to monitor patient adherence to the drug regime or to trace lost patients. Some experts also suggested the quality of HIV care services in private clinics might be poorer than in public clinics due to profit‐making needs.

However, some experts supported decentralization of ART into private clinics. It was envisaged that some HIV patients who could afford these services would prefer to use private clinics to reduce the chance of beings seen by others. On this basis, a plan to commence ART services in one private clinic in Jimma Town Health Office in collaboration with Regional Health Bureau gained some support from the panel. As one HIV programme manager observed:

We have heard that the district health office will start ART in a recognized private clinic… this is good and become as an alternative option. I know there are rich people who prefer to go to private clinics for collecting drugs. (Participant 13)

### 3.7 ART in health post

The panel members suggested that HEWs are not suitable to providing optimal ART service in the health post for the following reasons: low level of training, poor competency, poor ethical conducts, overload, job dissatisfaction, poor motivation and high turnover of health worker, unprofessionalism and issue of confidentiality. There is also a fear that HEWs may abuse the drugs. Even the health post setup may not be suitable, for example, having sufficient space for storing ART. Thus, from the patients’ perspectives and surrounding environment, ART in health posts may not work at this time. For example, an Associate Professor of Health Service Management said that:

ART in health posts is good as we bring the service to the community. However, HEWs are overburdened since everybody puts everything into them (hot potato syndrome …. laughing) …. (Participant 16)

Additionally, HIV programme managers in the sub‐regional (‘zone’) and district (‘woreda’) level did not agree that ART in health posts was a complicated issue. Citing the success of the community tuberculosis (Tb) management programme and maternal and child health services, they argued that ART in health posts could be successful. Capacity building and strict supervision would be preconditions to a roll‐out of an ART program, however. An HIV programme manager said,

Decentralization of ART service into health posts: I don’t think this is a difficult issue. We have seen this practically in community Tb program. So why not for HIV? My fear is, they may not have a capability to monitor the clinical and immunological managements. (Participant 11)

## 4 DISCUSSION

This study is the first of its kind in Ethiopia to contextually evaluate solutions for each cascade of HIV care by multi‐disciplined expert panellists. The panellists who comprised experts from diverse disciplines, expertise and professions ranked suggested solutions, discussed the strengths and weakness, and provided a range of opinions. For example, they suggested the inclusion of mandatory notification of partner's HIV status in a constitution. Under the constitution of the Federal Democratic Republic of Ethiopia, laws have been passed related to HIV. The Revised Family Code of July 2000 sets the need for HIV testing before marriage, and the revised criminal code (2005) prescribes punishment up to and including the death penalty for people who intentionally or knowingly spread HIV to others. Recognizing a gap in the existing constitution, the provision of a legal framework of mandatory notification of HIV status to a partner was supported by many of the experts, but its negative effects on HIV prevention and stigma was noted. It is obvious that if this legislation passes and people abide by it, it will contribute to the prevention of paternal, maternal and child health complications as partners would know each other's HIV status and probably use measure to prevent infecting the other. Globally, law about mandatory notification of HIV status to a partner are inconsistent in different countries and states. For example, in Australia, HIV‐positive patients are required to disclose their status before sexual contact in Tasmania and New South Wales, but not in the state of Victoria.^37^ In the United States, the penalty for non‐disclosure in North Carolina is stricter than in Alabama state.^38^ It seems difficult to enforce against traditional healers or religious curers who do not allow HIV patients to take their ART medicines into places of religious worship. Instead, we would suggest raising awareness in order for the religious healers who are trusted by many in the community to teach patients to take the pills while visiting the traditional healers simultaneously.

HIVST was also highly rated by the expert panel, and we believe that this programme can be very effective either alone or in combination with other programmes such as facility‐based HIV counselling or home‐based HIV testing. As literature informs, the uptake of this programme has been successful in similar settings,^39^ and our recommendation would be to support it as could be more acceptable when the oral test kits are available in Ethiopia. Additionally, other African countries including Malawi and Kenya have already introduced this programme stating that it is the key to achieving the first 90 (90% of all HIV patients knowing their status) of the UNAIDS target.^39^, ^40^, ^41^, ^42^ The use of case managers or peer educators, who are HIV patients themselves, in the HIV care was ranked in the top third of the seven suggested solutions. It is evident that involvement of HIV patients in HIV care is becoming an effective intervention to enhance testing coverage, ART linkage, ART retention and subsequently virological suppression. Different countries have found this programme to be very effective^43^, ^44^, ^45^ and the NGT expert panel also supported it.

The expert involved in the NGT did not support ART provision in selected private clinics in provinces in Ethiopia. Nonetheless, drawing from their experiences of implementing other programmes such as Tb programmes, local practitioners seemed to support this practice. In addition, it would provide an opportunity for a number of patients who would to access ART from private clinics, including in after hours. As a solution to providing option to patients who would like to access HIV treatment from private clinics, we suggest that a strict supportive supervision system be implemented by district health office and zonal health department. This provision should also be supported and referral linkages made with public hospitals. We are also reluctant to suggest decentralization of ART care services to the level of health post at its current situation due to among other things, inadequate infrastructure, lack of effective supervision and poor storage facilities for the ART. As well, the current levels of HEWs’ knowledge and competency would not support them to manage even minor complications following ART initiation. There are also frustrations with the HEWs in their commitment and job satisfaction and confidentiality. Although ART services exist in selected private hospitals, this is limited and only available in Addis Ababa, the capital city.^46^ However, other African countries such as South Africa and Cameron provide ART services in private clinics.^47^, ^48^

Regarding house‐to‐house HIV testing, even though the expert panellists supported the peer educators to run it, they recommended against the HEWs conducting home‐based HIV testing. As opposed to the expert panel, we suggest the home‐based HIV testing by peer educators in collaboration with or without HEWs. Confidentiality and stigma are people's main concern; however, additional capacity building can be provided for the HEWs on how to manage these and convince the community. From our qualitative exploration, we have observed that HEWs have successfully conducted HIV testing and family index HIV testing through the help of a local NGO. Although many solutions have been suggested by the current study, significant shortage of human resources for health in Ethiopia makes the HEW programme the only feasible option to conducting house‐to‐house HIV testing and subsequently achieve the UNAIDS target in HIV diagnosis. Additionally, home‐based HIV testing through community health workers or lay counsellors has been found to be effective in Southern Africa.^43^, ^49^

### 4.1 Limitations

The NGT of the present study has some limitations. Firstly, although experts suggested ideal recommendations known in theory, these could be less applicable in practice, the so‐called ‘self‐fulfillment prophecy’.^50^ Secondly, reaching consensus was an additional challenge in the NGT, a common limitation which has been noted in other consensus methods.^28^ Nevertheless, ultimate consensus was not the only aim, and our suggested solutions explored the minority suggestions to cater for feasibility in practice. Thirdly, even though we identified the expert panellists carefully, some disciplines, geographic areas and professions were possibly not as well represented as others. Fourthly, there was no statistical difference for the suggested solutions between the two rounds, and this could have been due to having compacted values of the Likert scale instrument.

## 5 CONCLUSIONS

The top three interventions rated by experts included mandatory notification of HIV status to partner, HIV self‐testing and the involvement of peer educators in the entire HIV care continuum. The combination of HIV self‐testing and facility‐based counselling is a plausible solution to address the downsides of the HIV self‐testing programme alone. The suggested solutions evaluated by the panel of experts are currently implemented in other African countries, and the current study gave a glimpse of the possibility to roll these programs out in Ethiopia. However, before considering to implement these interventions, a nationwide based study should be carried out using multi‐method approach.

## CONFLICT OF INTEREST

All authors declare that they have no competing interests.

## ETHICS APPROVAL

The NGT received ethical approvals from the Social and Behavioural Research Ethics Committee of Flinders University, South Australia (Project No: 7698) and the Institutional Review Board of Jimma University, Southwest Ethiopia (Ref. No: IHRPG/878/2017). We obtained written consent from each expert.

## Data Availability

The data that support the findings of this study are available from the corresponding author upon reasonable request.
